# The association between implementation strategy use and the uptake of hepatitis C treatment in a national sample

**DOI:** 10.1186/s13012-017-0588-6

**Published:** 2017-05-11

**Authors:** Shari S. Rogal, Vera Yakovchenko, Thomas J. Waltz, Byron J. Powell, JoAnn E. Kirchner, Enola K. Proctor, Rachel Gonzalez, Angela Park, David Ross, Timothy R. Morgan, Maggie Chartier, Matthew J. Chinman

**Affiliations:** 1 Center for Health Equity Research and Promotion, VA Pittsburgh Healthcare System, University Drive, Pittsburgh, PA 15240 USA; 2 Department of Surgery, University of Pittsburgh, Pittsburgh, PA USA; 3 Division of Gastroenterology, Hepatology, and Nutrition, University of Pittsburgh, Pittsburgh, PA USA; 4 Center for Healthcare Organization and Implementation Research, Edith Norse Rogers Memorial VA Hospital, Bedford, MA USA; 5 Department of Psychology, Eastern Michigan University, Ypsilanti, MI USA; 6 VA Center for Clinical Management Research, VA Ann Arbor Healthcare System, Ann Arbor, MI USA; 7 Department of Health Policy and Management, Gillings School of Global Public Health, University of North Carolina at Chapel Hill, Chapel Hill, NC USA; 8 Department of Veterans Affairs Medical Center, HSR&D and Mental Health Quality Enhancement Research Initiative (QUERI), Central Arkansas Veterans Healthcare System, Little Rock, AR USA; 9 Brown School, Washington University in St. Louis, St. Louis, MO USA; 10 Gastroenterology Section, VA Long Beach Healthcare System, Long Beach, CA USA; 11 New England Veterans Engineering Resource Center, VA Boston Healthcare System, Boston, MA USA; 12 HIV, Hepatitis and Related Conditions Programs, Office of Specialty Care Services, Veterans Health Administration, Washington, DC USA; 13 RAND Corporation, Pittsburgh, PA USA

**Keywords:** Interferon-free medications, Importance, Feasibility

## Abstract

**Background:**

Hepatitis C virus (HCV) is a common and highly morbid illness. New medications that have much higher cure rates have become the new evidence-based practice in the field. Understanding the implementation of these new medications nationally provides an opportunity to advance the understanding of the role of implementation strategies in clinical outcomes on a large scale. The Expert Recommendations for Implementing Change (ERIC) study defined discrete implementation strategies and clustered these strategies into groups. The present evaluation assessed the use of these strategies and clusters in the context of HCV treatment across the US Department of Veterans Affairs (VA), Veterans Health Administration, the largest provider of HCV care nationally.

**Methods:**

A 73-item survey was developed and sent to all VA sites treating HCV via electronic survey, to assess whether or not a site used each ERIC-defined implementation strategy related to employing the new HCV medication in 2014. VA national data regarding the number of Veterans starting on the new HCV medications at each site were collected. The associations between treatment starts and number and type of implementation strategies were assessed.

**Results:**

A total of 80 (62%) sites responded. Respondents endorsed an average of 25 ± 14 strategies. The number of treatment starts was positively correlated with the total number of strategies endorsed (*r* = 0.43, *p* < 0.001). Quartile of treatment starts was significantly associated with the number of strategies endorsed (*p* < 0.01), with the top quartile endorsing a median of 33 strategies, compared to 15 strategies in the lowest quartile. There were significant differences in the types of strategies endorsed by sites in the highest and lowest quartiles of treatment starts. Four of the 10 top strategies for sites in the top quartile had significant correlations with treatment starts compared to only 1 of the 10 top strategies in the bottom quartile sites. Overall, only 3 of the top 15 most frequently used strategies were associated with treatment.

**Conclusions:**

These results suggest that sites that used a greater number of implementation strategies were able to deliver more evidence-based treatment in HCV. The current assessment also demonstrates the feasibility of electronic self-reporting to evaluate ERIC strategies on a large scale. These results provide initial evidence for the clinical relevance of the ERIC strategies in a real-world implementation setting on a large scale. This is an initial step in identifying which strategies are associated with the uptake of evidence-based practices in nationwide healthcare systems.

## Background

A great deal of research now clearly shows that moving effective programs and practices into routine care settings requires the skillful use of implementation strategies, defined as “methods or techniques used to enhance the adoption, implementation, and sustainability of a clinical program or practice” [1]. Implementation strategies can vary widely and their labels can also vary. In order to generate a common nomenclature for implementation strategies and facilitate standardization of research methods in implementation science, the Expert Recommendations for Implementing Change (ERIC) study [2] engaged experts in modified-Delphi and concept mapping exercises to (1) refine a compilation of implementation strategies and (2) develop conceptually distinct categories of implementation strategies. This led to a compilation of 73 discrete implementation strategies (e.g., access new funding, audit and provide feedback, facilitation) [3], which were further organized into nine clusters [3]. These clusters include changing infrastructure, utilizing financial strategies, supporting clinicians, providing interactive assistance, training and educating stakeholders, adapting and tailoring to the context, developing stakeholder interrelationships, using evaluative and iterative strategies, and engaging consumers. This study is the first attempt to empirically determine whether these strategies and clusters of strategies are associated with the uptake of evidence-based practices (EBP) within the US Department of Veterans Affairs (VA), Veterans Health Administration (VHA).

Hepatitis C virus (HCV) infection is a leading cause of cirrhosis and liver cancer [4] in the USA and in VA. VA is the single largest provider of HCV care in the USA with approximately 174,000 Veterans who were potentially eligible for treatment in 2015 [5]. In the past, HCV treatment required interferon-based therapies, which involved long courses of injections and had numerous side effects and contraindications. These barriers resulted in only 23% of Veterans with HCV ever receiving treatment with these regimens [6]. Starting in December 2013, the first interferon-free drug combinations, Direct Acting Antivirals (DAAs), with substantially fewer side effects, shorter treatment course, and a higher cure rate, were FDA approved for specific genotypes of HCV. By October 2014 or the start of fiscal year (FY) 2015, interferon-free combinations were available for all genotypes of HCV, making them the new evidence-based practice for treating HCV [7–13]. However, these innovative treatments and their high costs posed a significant challenge to VA, requiring the healthcare system to adjust policies, resource availability, and staffing [6].

Reaching and treating Veterans infected with HCV required significant restructuring to more rapidly deliver medications, expand the reach of treatment to Veterans who were previously ineligible, and bring Veterans into care who were not previously engaged in care. In order to accomplish these goals and facilitate the uptake of the EBP, VA developed a novel program, the Hepatitis C Innovation Team Collaborative (HIT). In FY 2015, all 21 regional administrative centers, or Veterans Integrated Service Networks (VISNs), were directed to form multidisciplinary teams. Team members represented multiple sites (e.g., medical centers and outpatient clinics) within their region. Teams were both financially and organizationally supported to develop strategies, utilizing Lean principles of quality improvement [14], to increase treatment rates and improve access to and quality of HCV care. The engagement of local providers in the Collaborative varied by site, and despite the centralized support of providers, local providers were free to select implementation strategies. The focus of this assessment was on understanding which strategies were chosen and the impact of these strategies on treatment outcomes.

The availability of interferon-free HCV treatments on the national VA pharmacy formulary, sufficient funding to provide broad access to these medications, and local flexibility to choose implementation strategies across the VHA represented a unique laboratory in which to understand how a variety of implementation strategies affected the uptake of a highly evidence-based innovation. We hypothesized that the number of implementation strategies endorsed would be associated with increased uptake of the innovation (i.e., increased starts of interferon-free medications).

## Methods

### Overview

We assessed the uptake of strategies as defined by the ERIC project [2]. To develop the implementation assessment, the original 73 ERIC strategy descriptions were tailored to interferon-free treatment in VA and the strategies were organized by cluster. The survey was iteratively vetted by HIT leader stakeholders, five HCV treatment providers, and a psychometrician for readability and understandability. Care was taken to ensure fidelity to the original, previously defined strategies. Table 1 shows the questions in order of presentation organized by cluster. The survey asked about strategies used in fiscal year 2015 (FY15) to increase interferon-free HCV treatment at their VA medical center (VAMC). For each strategy, participants were asked, “Did you use X strategy to promote HCV care in your center?” The questions were anchored to FY15 so they could be linked to treatment data over the same time period. The Pittsburgh VA IRB determined that the initiative was exempt under a provision applying to quality improvement. This assessment was approved as a quality improvement project by the VA’s HIV, Hepatitis and Related Conditions Programs in the Office of Specialty Care Services as a part of the HIT Collaborative evaluation. All participation was completely voluntary.

**Table 1:** Strategies by cluster and correlation with treatment starts

No.	Strategy	Sites N (%)	Correlation	*P* value
	*In FY15 did your center use any of these infrastructure changes to promote HCV care in your center?*			
*1*	*Change physical structure and equipment (e.g., purchase a FibroScan, expand clinic space, open new clinics)*	*42 (53)*	*0.36*	*<0.01*
2	Change the record systems (e.g., locally create new or update to national clinical reminder in CPRS, develop standardized note templates)	57 (71)	−0.02	0.89
*3*	*Change the location of clinical service sites (e.g., extend HCV care to the CBOCs)*	*21 (26)*	*0.36*	*<0.01*
4	Develop a separate organization or group responsible for disseminating HCV care (outside of the HIT Collaborative)	18 *(23)*	0.21	0.07
5	Mandate changes to HCV care (e.g., when you changed to the new HCV medications was this based on a leadership mandate?)	44 (55)	0.05	0.69
6	Create or change credentialing and/or licensure standards (e.g., change scopes of practice or service agreements)	23 (29)	0.01	0.92
*7*	*Participate in liability reform efforts that make clinicians more willing to deliver the clinical innovation*	*3 (4)*	*0.23*	*0.04*
*8*	*Change accreditation or membership requirements*	*3 (4)*	*0.23*	*0.04*
	*In FY15 did your center use any of these financial strategies to promote HCV care in your center?*			
9	Access new funding (This DOES NOT include funding from national VA for the medications, but should include receiving funds from the HIT Collaborative to your center)	24 *(30)*	0.20	0.08
10	Alter incentive/allowance structures	4 *(5)*	0.04	0.76
11	Provide financial disincentives for failure to implement or use the clinical innovations	0	.	.
12	Respond to proposals to deliver HCV care (e.g., submit a HIT proposal to obtain money for your center specifically)	35 *(44)*	0.19	0.11
13	Change billing (e.g., create new clinic codes for billing for HCV treatment or HCV education)	9 *(11)*	0.17	0.15
14	Place HCV medications on the formulary	56 *(70)*	−0.05	0.67
15	Alter patient fees	0		
16	Use capitated payments	0		
17	Use other payment schemes	4 *(5)*	0.22	0.06
*18*	*Create new clinical teams (e.g., interdisciplinary clinical working groups)*	*37 (46)*	*0.25*	*0.04*
19	Facilitate the relay of clinical data to providers (e.g., provide outcome data to providers)	45 *(56)*	0.20	0.09
*20*	*Revise professional roles (e.g., allow the pharmacist to see and treat patients in the clinic)*	*57 (71)*	*0.24*	*0.04*
21	Develop reminder systems for clinicians (e.g., use CPRS reminders)	27 *(34)*	−0.16	0.19
*22*	*Develop resource sharing agreements (e.g., partner with the VERC, the HITs, or other organizations with the resources to help implement changes)*	*21 (26)*	*0.24*	*0.04*
	*In FY15 did your center employ any of these activities to provide interactive assistance to promote HCV care in your center?*			
23	Use outside assistance often called “facilitation” (e.g., coaching, education, and/or feedback from the facilitator)	6 *(8)*	0.16	0.17
*24*	*Have someone from inside the clinic or center (often called “local technical assistance”) tasked with assisting the clinic*	*12 (15)*	*0.38*	*<0.01*
*25*	*Provide clinical supervision (e.g., train providers)*	*35 (44)*	*0.29*	*0.01*
*26*	*Use a centralized system (i.e., from the VISN) to deliver facilitation*	*22 (28)*	*0.38*	*<0.01*
	*In FY15 did your center employ any of these activities to tailor HCV care in your center?*			
27	Use data experts to manage HCV data (e.g., use the VERC, pharmacy benefits management, VISN, or CCR data experts to track patients or promote care)	46 *(58)*	0.18	0.12
28	Use data warehousing techniques (e.g., dashboard, clinical case registry, CDW)	68 *(85)*	0.15	0.19
29	Tailor strategies to deliver HCV care (i.e., alter HCV care to address barriers to care that you identified in your population using data you collected)	50 *(63)*	0.21	0.08
30	Promote adaptability (i.e., Identify the ways HCV care can be tailored to meet local needs and clarify which elements of care must be maintained to preserve fidelity)	44 *(55)*	0.16	0.17
	*In FY15 did your center employ any of these activities to train or educate providers to promote HCV care in your center?*			
*31*	*Conduct educational meetings*	*41 (51)*	*0.24*	*0.05*
*32*	*Have an expert in HCV care meet with providers to educate them*	*33 (41)*	*0.34*	*<0.01*
*33*	*Provide ongoing HCV training*	*39 (49)*	*0.26*	*0.03*
*34*	*Facilitate the formation of groups of providers and fostered a collaborative learning environment*	*35 (44)*	*0.38*	*<0.01*
35	Developed formal educational materials	31 *(39)*	0.00	0.97
36	Distribute educational materials (e.g., guidelines, manuals, or toolkits)	44 *(55)*	0.11	0.35
37	Provide ongoing consultation with one or more HCV treatment experts	46 *(58)*	0.11	0.37
38	Train designated clinicians to train others (e.g., primary care providers, SCAN-ECHO)	16 *(20)*	−0.07	0.56
*39*	*Vary the information delivery methods to cater to different learning styles when presenting new information*	*29 (36)*	*0.29*	*0.02*
40	Give providers opportunities to shadow other experts in HCV	26 *(33)*	0.12	0.32
41	Use educational institutions to train clinicians	9 *(11)*	0.21	0.07
	*In FY15 did your center employ any of these activities to develop stakeholder interrelationships to promote HCV care in your center?*			
*42*	*Build a local coalition/team to address challenges*	*42 (53)*	*0.27*	*0.03*
*43*	*Conduct local consensus discussions (i.e., determine how to change things by having meetings with local leaders and providers)*	*38 (48)*	*0.42*	*<0.01*
44	Obtain formal written commitments from key partners that state what they will do to implement HCV care (e.g., written agreements with CBOCS)	3 *(4)*	0.20	0.09
*45*	*Recruit, designate, and/or train leaders*	*21 (26)*	*0.29*	*0.01*
*46*	*Inform local opinion leaders about advances in HCV care*	*39 (49)*	*0.33*	*<0.01*
*47*	*Share the knowledge gained from quality improvement efforts with other sites outside your medical center*	*30 (38)*	*0.32*	*<0.01*
*48*	*Identify and prepare champions (i.e., select key individuals who will dedicate themselves to promoting HCV care)*	*40 (50)*	*0.29*	*0.01*
49	Organize support teams of clinicians who are caring for patients with HCV and given them time to share the lessons learned and support one another’s learning	21 (26)	0.16	0.18
50	Use advisory boards and interdisciplinary workgroups to provide input into HCV policies and elicit recommendations	21 (26)	0.09	0.46
51	Seek the guidance of experts in implementation	35 (44)	−0.01	0.92
*52*	*Build on existing high-quality working relationships and networks to promote information sharing and problem solving related to implementing HCV care*	*49* (61)	*0.24*	*0.04*
*53*	*Use modeling or simulated change*	*10* (13)	*0.25*	*0.04*
*54*	*Partner with a university to share ideas*	*11 (14)*	*0.27*	*0.02*
*55*	*Make efforts to identify early adopters to learn from their experiences*	*13 (16)*	*0.32*	*<0.01*
*56*	*Visit other sites outside your medical center to try to learn from their experiences*	*12 (15)*	*0.30*	*0.01*
57	Develop an implementation glossary	2 *(3)*	0.17	0.15
58	Involve executive boards	18 *(23)*	0.15	0.21
	*In FY15 did your center employ any of these evaluative and iterative strategies to promote HCV care in your center?*	*2 (3)*		
59	Assess for readiness and identify barriers and facilitators to change (e.g., administer the organizational readiness to change survey)	21 *(26)*	0.16	0.20
60	Conduct a local needs assessment (i.e., collect data to determine how best to change things)	36 *(45)*	0.12	0.31
61	Develop a formal implementation blueprint (i.e., make a written plan of goals and strategies)	27 *(34)*	0.11	0.37
62	Start with small pilot studies and then scale them up	18 *(23)*	0.08	0.50
*63*	*Collect and summarize clinical performance data and give it to clinicians and administrators to implement changes in a cyclical fashion using small tests of change before making system-wide changes*	*17 (21)*	*0.25*	*0.04*
64	Conduct small tests of change, measured outcomes, and then refined these tests	15 *(19)*	0.11	0.36
65	Develop and use tools for quality monitoring (this includes standards, protocols and measures to monitor quality)	33 *(41)*	0.07	0.56
66	Develop and organize systems that monitor clinical processes and/or outcomes for the purpose of quality assurance and improvement (i.e., create an overall system for monitoring quality--not just tools to use in quality monitoring, which is addressed in the last item)	24 *(30)*	0.18	0.14
67	Intentionally examine the efforts to promote HCV care	49 *(61)*	0.08	0.49
68	Develop strategies to obtain and use patient and family feedback	16 *(20)*	−0.11	0.35
	*In FY15 did your center employ any of these strategies to engage patient consumers to promote HCV care in your center?*			
69	Involve patients/consumers and family members	40 *(50)*	0.01	0.91
*70*	*Engage in efforts to prepare patients to be active participants in HCV care (e.g., conduct education sessions to teach patients about what questions to ask about HCV treatment)*	*50 (63)*	*0.39*	*<0.01*
71	Intervene with patients/consumers to promote uptake and adherence to HCV treatment	57 (71)	0.08	0.51
72	Use mass media (e.g., local public service announcements; magazines like VANGUARD, newsletters, online/social media outlets) to reach large numbers of people	14 (18)	0.00	0.98
73	Promote demand for HCV care among patients through any other means	32 (40)	0.19	0.12

Statistically significant strategies are represented in *italics*

### Participation sites and recruitment

The HIT Collaborative provided the contact information for VA HCV providers and HIT members representing all 130 individual VA sites. The sites, for the purpose of this assessment, were defined as all VA medical “stations” as classified by Population Health Services of the VA. These stations include a larger medical center, and some of these stations have smaller satellite sites. All sites within a station would be included in the measures of treatment starts for the station. However, most of the treatment starts are coordinated by and occur in the larger medical center within these stations. This assessment included all stations, herein deemed “sites,” regardless of participation in the HIT Collaborative. Respondents were surveyed via an email link to an online survey portal. A modified Dillman approach [15] was used to promote high response, with two mass emails and one individual email to reach potential participants. In order to maximize survey completion rates, the recruitment email was cosigned by the HIT Collaborative Leadership team. Additionally, the Leadership Team provided coordinated communication with HIT members on regularly scheduled calls so that providers were aware of the assessment and its purpose.

At sites with multiple respondents, we assessed interrater reliability but ultimately retained one respondent per site. The retention of one respondent was based on the “key informant” technique, [16] in part to reduce bias of increased reporting from the sites with duplicate responses. If an HCV lead clinician, designated by the VAMC, was available and responded, their answer was retained; otherwise, if there were multiple respondents, they were prioritized by who would know the most about HCV treatment in the following order (established a priori): physician, pharmacist, advanced practice provider (nurse practitioner or physician assistant), other provider, and system redesign staff.

### Measures and data collection

The primary outcome of interest was the number of Veterans started on the new interferon-free medications for HCV during FY15 from each VAMC (deemed “treatment starts”), which was obtained from VA’s population health intranet database [5]. A secondary outcome was the proportion of viremic patients so treated, assessed by dividing the number of patients started on the medications by the number of Veterans with known active HCV infection in need of treatment at each site. VA uses a Clinical Case Registry to validate HCV cases at each site, and the numbers are reported nationally. A local coordinator is sent the results of patients who are found to be HCV positive and the coordinator at that site determines whether the patient truly has HCV. Veterans are considered to be viremic if they have a positive viral load on their most recent testing, are alive at the end of the year of interest, and have been validated as HCV positive through the CCR. In order to be a part of a site’s patient load, they had to be considered “in care,” meaning that they needed to have had an encounter or medication fill within the prior 365 days at that medical facility. Descriptive statistics were used to describe the frequency of strategy and cluster endorsement and the association between strategy use and medication starts. Data were analyzed as follows: (1) the total number of strategies and (2) the number of strategies within a cluster. As described in Waltz et al. [17], strategies were grouped into quadrants via combinations of importance (i.e., how vital a strategy was rated to be in improving implementation, grouped into low and high categories) and feasibility (i.e., how possible a certain strategy is to do, also grouped in to low and high categories). Using the definitions per Waltz et al., these quadrants included high feasibility/high importance strategies (quadrant 1), low importance/high feasibility (quadrant 2), low importance/low feasibility (quadrant 3), and high importance/low feasibility (quadrant 4) [3].

A key covariate used in the analyses is “site complexity.” Site complexity in VA was assessed using ratings from the VHA Facility Complexity Model. First created in 1989 and regularly updated [18], the ratings combine site level of care acuity, services available, research dollars, and patients served. The ratings include standardized classifications into levels 1a, 1b, 1c, 2, and 3, in decreasing levels of complexity. Thus, level 1a facilities have high volume, high-risk patients, the most complex clinical programs, and largest teaching and research programs, and level 3 programs have low volume, low-risk patients, few or no complex clinical programs, and small or no research or teaching programs [18]. We had conducted a prior survey of the programs treating HCV, asking respondents to self-report the number of providers treating HCV at each VA site. These data were added to the dataset in order to determine whether staffing related to treatment starts and the proportion of viremic patients treated.

### Data analysis

All analyses were conducted using the R statistical package. Non-parametric statistical tests were used to assess the associations between dependent and independent variables including Wilcoxon rank-sum testing for treatment by strategy and Spearman’s correlation testing for continuous variables. Interrater reliability was calculated for sites with duplicate responses. We analyzed whether treatment was associated with using high-feasibility or high-importance strategies and also strategies in the “Go-zone” of high feasibility and importance (zone 1). A multivariable linear regression model was made to assess whether treatment starts were associated with implementation strategies, controlling for facility characteristics.

## Results

Of 130 unique stations that are engaged in HCV treatment in VA contacted for the assessment, 80 provided responses (62%). These 80 sites were responsible for 68% of national HCV treatment starts in FY15 (*n* = 20,503). A total of 133 responses were obtained; 53 were omitted from further analysis (15 opened the survey and did not respond to any questions, 29 were duplicate responses from 19 sites, and 9 did not list an associated VAMC and could not be linked to outcomes). Among responses within 19 duplicate sites, interrater reliability was 0.66. All 21 VA-defined regions of the country (VISNs, as defined in FY15) were represented. Table 2 illustrates respondent characteristics and shows that respondents were predominantly from specialty services of gastroenterology/hepatology or infectious disease. Pharmacists made up the largest group of respondents. There were no significant associations between participant characteristics and treatment starts or number of strategies used. The majority of sites represented were complexity level 1 (Table 2). In addition to standard complexity scoring, we analyzed the number of providers treating HCV during the FY of interest from a prior assessment. These data were available for 49 of the sites (61%). The median number of providers treating HCV in these sites was 5 (3,7), including physicians, advanced practitioners, pharmacists, and nurses.

**Table 2:** Respondent characteristics

Characteristic	N (sites)	Percentage
Years in VA		
<3	13	16
4 to 9	25	31
10 to 19	25	31
>20	17	21
Specialty		
Gastroenterology/hepatology	33	41
Infectious disease	17	21
Pharmacy	13	16
Primary care	8	10
Other (VERC, transplant)	9	11
Degree		
PharmD	35	44
NP	13	16
MD	11	14
PA	5	6
RN	2	3
Other	14	18
Site complexity		
1a	27	33
1b	14	18
1c	12	15
2	14	18
3	12	15

Among responding sites, the median number of treatment starts was 197 (IQR = 124, 312). The median number of Veterans potentially eligible for treatment at the start of FY15 at these sites was 1149 (IQR = 636, 1744). The proportion of viremic patients treated ranged from 6 to 47%, with a mean (SD) of 20 ± 8%. The sites that did not respond had a median number of treatment starts of 142 (IQR = 88, 296), which was not significantly different from responding sites (*p* = 0.07). The median number of Veterans potentially eligible for treatment at the start of FY15 at these non-responding sites was 874 (IQR = 494, 1716), which was not significantly different than responding sites (*p* = 0.43). The proportion of viremic patients treated ranged from <1–46%, with a mean of 17 ± 10%, which was not statistically different than responding sites (*p* = 0.15).

Table 1 shows the strategies in order of presentation on the survey with the questions as they were asked. Sites endorsed between 1 and 59 strategies, with an average of 25 ± 14. Quartile of treatment starts was significantly associated with the number of strategies endorsed (*p* < 0.01), with the top quartile endorsing a median of 33 strategies, compared to 15 strategies in the lowest quartile. Table 1 shows the frequency of the strategy endorsement, and the association between strategies and treatment starts. The most frequently used strategies included data warehousing techniques, (e.g., using a dashboard; 85%), and intervening with patients to promote uptake and adherence to HCV treatment (71%).

A total of 28 of the 73 specific strategies were associated with treatment starts (Table 1, in bold). Associations between treatment starts and strategies were assessed with correlation coefficients and Wilcoxon rank-sum tests in order to assess for stability of results. Notably, the strategies that were significant were consistent regardless of the type of statistical test applied. Sites using at least 15 of these significant strategies (*n* = 20) had increased median treatment starts (320 vs. 177, *p* < 0.001). There was a moderate, positive correlation between overall total number of strategies and treatment starts, which was statistically significant (*r* = 0.43, *p* < 0.001). The strategies most correlated with medication initiations were “conduct local consensus discussions” (0.42, *p* < 0.001), “engage in efforts to prepare patients to be active participants in HCV care” (0.39, *p* < 0.001), “facilitate the formation of groups of providers and [foster] a collaborative learning environment” (0.38, *p* = 0.001), “use a centralized system to deliver facilitation” (0.38, *p* = 0.001), “have someone from inside the clinic or center (often called local technical assistance) tasked with assisting the clinic” (0.38, *p* = 0.001), “revise professional roles” (0.36, *p* = 0.053), “conduct local consensus discussions” (0.36, *p* = 0.002), “change physical structure and equipment” (0.36, *p* = 0.002), and “change location of clinical service sites” (0.36, *p* = 0.001). The strategies were from different clusters, though there were two strategies each from the interactive assistance and infrastructure change clusters.

In order to assess the impact of strategies on treatment starts while accounting for facility characteristics, a multivariable model was made assessing whether the number of strategies used remained associated with treatment while controlling for facility complexity. In this regression model, facility complexity and number of strategies were both significantly associated with treatment starts. The adjusted *R* ^2^ for the model was 30%.

**Fig. 1:**
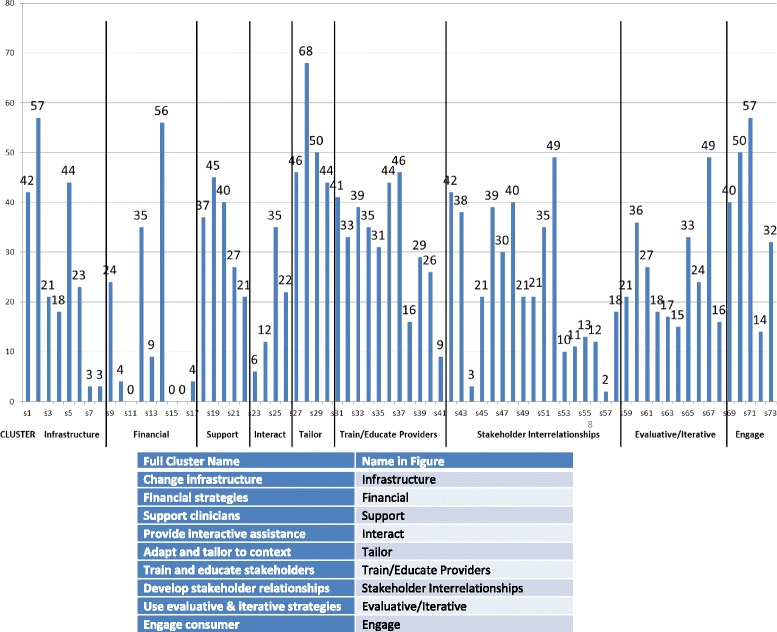
Endorsement of strategies by cluster

The individual strategies were assessed by cluster to determine the relationships between clusters of strategies and treatment starts. Figure 1 shows the frequency with which strategies were endorsed by cluster.

Table 3 shows that the number of strategies used within a cluster was also significantly associated with treatment starts for each individual cluster. The clusters with the highest proportion of significant strategies were “provide interactive assistance” (75% of strategies significantly associated with treatment) and “develop stakeholder interrelationships” (64% of strategies significantly associated with treatment) and those with the least were “tailor to the context” and “financial strategies.” None of the individual financial strategies or tailoring strategies were associated with treatment starts. Otherwise, there was at least one strategy significantly correlated with number of treatment starts in every cluster (Table 1).

**Table 3:** Correlation between items endorsed in the cluster and treatment starts

Implementation strategy clusters	Number of strategies	Number of Endorsements (number per strategy in cluster)	Correlation between number of strategies used within the cluster and treatment starts	*R* ^2^	*P* value	Number (%) of strategies in the cluster associated with treatment starts
Provide interactive assistance	4	75 (19)	0.46	21%	<0.001	3 (75%)
Develop stakeholder relationships	17	405 (24)	0.44	20%	<0.001	11 (64%)
Train and educate stakeholders	11	349 (32)	0.33	11%	0.003	5 (45%)
Adapt and tailor to context	4	208 (52)	0.31	10%	0.004	0 (0%)
Change infrastructure	8	211 (26)	0.29	9%	0.008	4 (50%)
Support clinicians	5	187 (37)	0.29	8%	0.009	3 (60%)
Engage consumer	5	193 (39)	0.27	7%	0.016	1 (20%)
Financial strategies	9	141 (16)	0.26	7%	0.020	0 (0%)
Use evaluative and iterative strategies	10	191 (19)	0.23	5%	0.043	1 (10%)

Figure 1 graphically depicts the density of endorsement across clusters. Notably, the most commonly used strategies were not those most strongly associated with treatment starts. While “providing interactive assistance” was the most strongly associated with treatment, this was among the least-endorsed clusters. Conversely, “adapting and tailoring to the context” was among the most highly and densely endorsed clusters but there were no strategies within this cluster associated with treatment.

Waltz et al. grouped strategies into quadrants by perceived feasibility (low/high) and importance (low/high), and a composite score [17]. Table 4 demonstrates how strategies in these quadrants were used by respondents. Multiple strategies were applied from each quadrant indicating that perceived low feasibility, as defined in the ERIC project, was not a barrier to reported uptake of strategies. Treatment starts were associated with the number of strategies in all quadrants. The number of both “high feasibility” and “high importance” strategies endorsed by sites were associated with number of treatment starts (*r* = 0.37, *p* < 0.001 and *r* = 0.39, *p* < 0.001, respectively). However, having a higher proportion of strategies from the “high importance” or “high feasibility” groups was not associated with higher treatment rates. In fact, the raw correlation coefficients were higher between treatment starts and the low feasibility quadrants’ association with treatment starts than for the high feasibility quadrants.

**Table 4:** Quadrant assessment

Quadrant	Description	Number of strategies in quadrant	Number of endorsements of strategies in quadrant by respondents	Endorsements per strategy	Number of strategies associated with treatment starts in quadrant (% of strategies in quadrant)	Correlation between number strategies used in quadrant and treatment starts *r* (*p*)	Correlation between number strategies used in quadrant and number viremic *r* (*p*)
1	High importance, high feasibility	31	966	31	10 (32%)	0.35 (0.002)	0.38 (<0.001)
2	Low importance, high feasibility	11	215	20	5 (45%)	0.37 (<0.001)	0.38 (<0.001)
3	Low importance, low feasibility	22	542	25	9 (41%)	0.44 (<0.001)	0.37 (<0.001)
4	High importance, low feasibility	9	293	33	4 (44%)	0.44 (<0.001)	0.41 (<0.001)

Table 5 illustrates the strategies that were most commonly endorsed among sites in the high and low quartiles of treatment starts. Five of the strategies were shared between the groups (denoted in red in the table), and five were distinct between the groups of sites. There were differences in the strategies used by high and low quartile sites: low quartile sites were more likely to endorse “mandating change” and “changing the record system” while high quartile sites were more likely to “change equipment and physical structures” as well as to “facilitate relay of clinical data to providers.”

**Table 5:** Most commonly used strategies in the top and bottom quartile of treatment starts

Top treating quartile	Cluster	N	Quadrant	Bottom treating quartile	Cluster	*N*	Quadrant
Revise professional roles^a^	Support clinicians	14	3	Intentionally examine the efforts to promote HCV care	Evaluative	9	1
Identify and prepare champions^a^	Interrelationships	14	1	Place HCV medications on the formulary	Financial	13	4
Tailor strategies to deliver HCV care	Tailor	15	1	Provide ongoing consultation with one or more HCV treatment experts	Train/educate	9	1
Engage in efforts to prepare patients to be active participants in HCV care^a^	Consumers	16	4	Mandate changes to HCV care	Infrastructure	13	3
Change the record systems	Infrastructure	14	3	Develop reminder systems for clinicians	Support	9	2
Intervene with patients/consumers to promote uptake and adherence to HCV treatment	Consumers	17	4	Intervene with patients/consumers to promote uptake and adherence to HCV treatment	Consumers	14	4
Use data warehousing techniques	Tailor	19	3	Use data warehousing techniques	Tailor	16	3
Distribute educational materials	Train/educate	14	1	Distribute educational materials	Train/educate	9	1
Facilitate the relay of clinical data to providers	Support	15	1	Facilitate the relay of clinical data to providers	Support	11	1
Build on existing high-quality working relationships and networks to promote information sharing and problem solving related to implementing HCV care^a^	Interrelationships	15	3	Build on existing high-quality working relationships and networks to promote information sharing and problem solving related to implementing HCV care^a^	Interrelationships	9	3

^a^Strategies significantly correlated with treatment starts (see Table 2)

In addition to assessing treatment starts, the proportion of viremic patients treated was assessed. This measure was not significantly associated with use of any particular strategy or cluster, number of strategies used overall, or with facility complexity or number of providers.

**Fig. 2:**
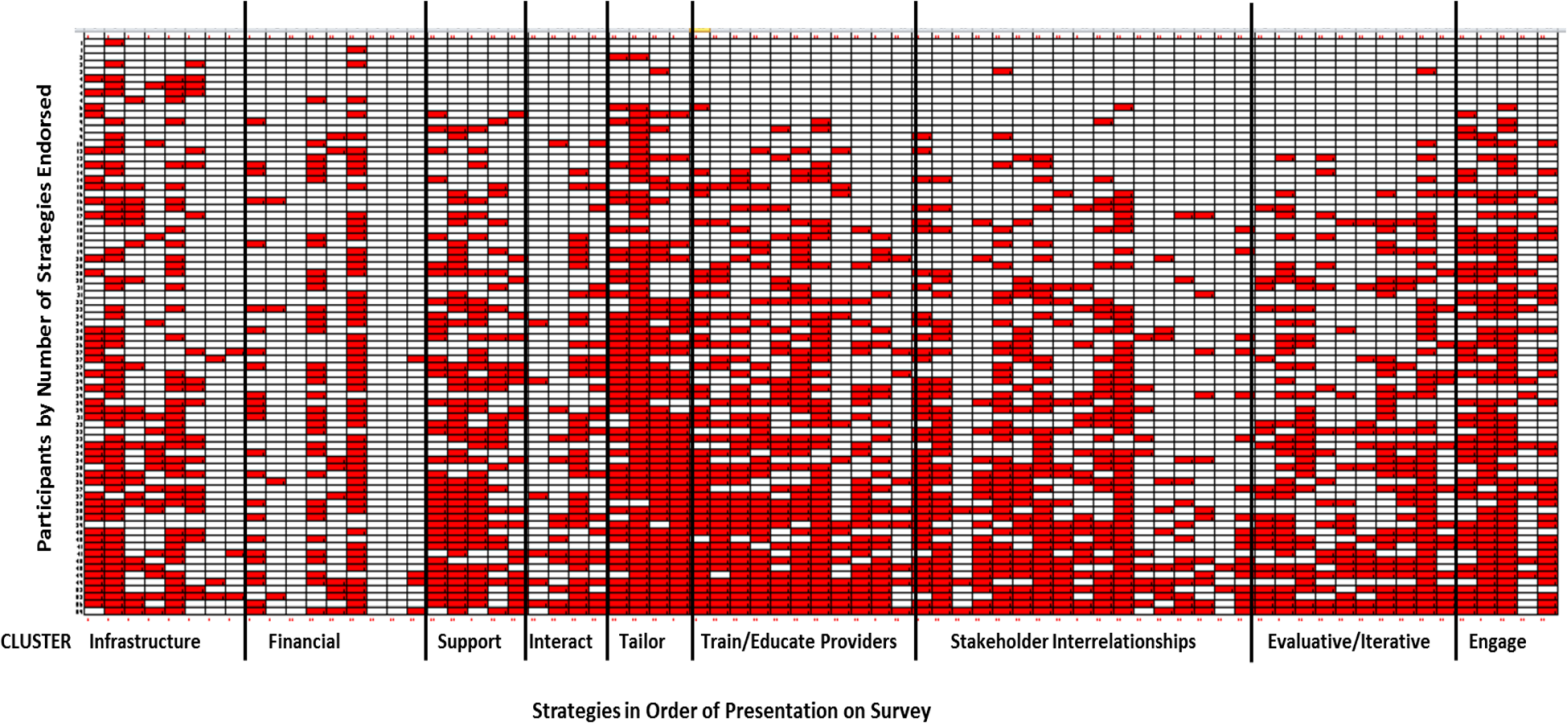
Strategies in order of presentation on the survey by participant. This density plot represents strategy endorsements made by each participant in the order in which they were presented in the survey from *left* to *right*. Respondents, represented by *rows*, were sorted by the number of strategies they endorsed with those endorsing the least at the top of the plot and those endorsing the most at the *bottom*

Figure 2 illustrates graphically that there was no drop-off in participation as the assessment progressed, by illustrating the responses by number of strategies endorsed and by order of the assessment. Participants endorsed as many items at the end of the survey as at the start of the survey, including participants who endorsed <5 items. This suggests that there was not a bias towards selecting items earlier in the assessment. This figure also graphically illustrates the density of endorsement by strategy and cluster.

## Discussion

Implementation strategies that VA medical centers used to promote HCV treatment with interferon-free medications were assessed based on the nomenclature developed in the ERIC study. Specifically, we explored whether the ERIC strategies and clusters of strategies were associated with implementation of an innovative evidence-based practice, in this case the use of interferon-free medications for HCV. The use of interferon-free medications for HCV is highly evidence-based, is uniformly and simply applied, widely implemented, and easily documented and extractable from the medical record in a reliable fashion. This makes the use of interferon-free medications for HCV an ideal case in which to understand how implementation strategies function in a real-world context on a large scale. The presented data suggest that the ERIC strategies are empirically related to the use of interferon-free medications in a large, nationwide, scale-up effort to increase the uptake of this evidence-based practice. These data support the hypothesis that the use of more strategies is associated with increased use of interferon-free medications for HCV.

However, the most commonly used strategies and clusters of strategies were not those associated with the highest number of treatment starts. Only 3 of the top-endorsed 15 strategies were correlated with treatment starts. These included the following strategies: “revise professional roles,” “build on existing high-quality working relationships and networks to promote information sharing and problem solving,” and “engage in efforts to prepare patients to be active participants.” Whether the 12 non-correlated strategies serve meaningful supportive roles for those strategies that do correlate with treatment starts (i.e., that they may be necessary but not sufficient) is unknown. The clusters with the highest percentage of strategies associated with treatment rates were “providing interactive assistance,” “supporting clinicians,” and “developing stakeholder interrelationships.” The importance of these kinds of interactive and supportive strategies is consistent with the literature on implementation “facilitation,” in which outside aid is provided to help sites with a wide range of activities needed to start new initiatives [19]. Overall sites used multiple strategies including those that were previously identified as less feasible by implementation researchers and clinical managers [3]. In fact, the two “low feasibility” quadrants (both high and low importance) had numerically higher correlations with treatment starts than the “high feasibility” quadrants. It is possible that the sites able to conduct more difficult and complex (i.e., less feasible) implementations strategies, including interactive assistance, were able to treat more patients. Additionally, these findings may indicate that feasibility and importance could vary across innovation, context, and recipients. An alternative explanation is that the feasibility of strategies as identified in a theoretical process (as was ERIC) does not accurately reflect what is applied in routine clinical implementation. Future research may need to assess how perceptions of feasibility and importance operate across different clinical contexts.

The data regarding successful strategies are consistent with prior implementation research regarding efficacy of specific strategies. For example, “mandating change” was a strategy often endorsed by the sites in the lowest quartile of treatment and not by the higher treating sites, which is consistent with prior evidence for the minimal effectiveness of top-down mandates in implementing change [20, 21]. Overall, five of the top ten strategies for the top and bottom quartiles were shared by both groups. The sites in the highest quartile of treatment tended to use more interpersonally focused strategies (3 of 5 distinct strategies including “revising professional roles,” “preparing champions,” and “preparing patients”) while only 1 of the 5 distinct strategies in the lowest quartile of treatment was interpersonally focused (e.g., “consultation”). However, there were not specific clusters or quadrants that predicted being in the highest vs. lowest quartile of treatment.

None of the strategies within the financial cluster were significantly associated with treatment starts. There are a variety of financial strategies and several are not applicable within VA. For example, sites do not have the flexibility to change billing, provide financial incentives, use capitated payments, or alter patient fees. Sites do have the ability to create new clinic codes, for example, if pharmacists have new clinics, and a few sites endorsed this item. “Accessing new funding” via grants or the HIT program and “responding to proposals” were items that were frequently endorsed by sites. These were not associated with treatment starts, possibly because the financial component provided by the HIT was modest. One financial strategy of note was adding medications to the formulary. VA fiscal and formulary changes are nationally applied. Thus, the HCV medications were placed on the national formulary at the same time for all sites. However, it is notable that the low treating sites were more likely to endorse “placing HCV medications on the formulary” as among their most frequently-used strategies. This is likely a reflection of the lack of other active local strategies being used in these sites. These sites were more likely to attribute the national formulary change to the actions of their site than the sites that were more active in their implementation.

It is possible that an association between the strategies used and treatment starts could be due simply to facility characteristics. For example, larger sites may have more capacity in the form of staffing and resources to engage in treatment. However, this was not found in this sample. The number of staff was not significantly associated with the number of treatment starts. Similarly, the number of providers was not significantly correlated with the number of strategies or the number of significant strategies. More complex medical centers were more likely to choose the strategies that were significantly associated with treatment starts. Future work should focus on the interactions between the types of strategies used and facility complexity.

This assessment was an example of adapting the ERIC strategies to a specific clinical issue. While the survey items maintained fidelity to each of the 73 implementation strategies in ERIC, they were also tailored to HCV with specific examples. The process required vetting the survey with stakeholders to ensure that the examples were relevant and understandable. Presenting complex implementation terms in long lists to stakeholders was found to be feasible with a reasonable response rate in this national sample. The high interrater reliability among participants from sites with multiple respondents suggests that participants are interpreting the strategies consistently within the sites. However, more qualitative work will be required to determine how community stakeholders do or do not distinguish between particular strategies. This general approach of using a structured survey could be used to track strategy use over time in implementation research and practice. Failing to accurately track strategy use limits abilities to explain how and why specific efforts succeed or fail. This type of approach could move implementation science towards a more comprehensive understanding of how various strategies operate across settings and disease states. There is currently a paucity of measures to assess implementation strategy use [22] and this study contributes to the literature by providing one approach to assess strategy use through an online survey.

There were several limitations of this evaluation. Despite the fact that this was a national assessment with high response rates, the total number of respondents was small compared to the number of strategies that were investigated, which limited the power to assess multiple variables in the same models. This was a cross-sectional assessment and future assessments will allow us to better understand longitudinal associations of the strategies with treatment rates over time. Over FY15, there were multiple national policy shifts that dramatically and transiently impacted funding for HCV treatment. These changes likely impacted treatment rates, independent of implementation strategy use. However, given that these changes occurred on a national level secular changes would theoretically affect sites uniformly. In this investigation we were unable to assess interactions between policy changes and implementation strategies, though this would be of interest. While these assessments within VA allowed us to examine these implementation strategies in a national system, the external validity in a non-VA system will need to be investigated in future studies. The effects of patient factors, particularly gender, will also need to be assessed, given the predominance of men in the VA system. While we chose a key informant technique, using one respondent per center, and had high interrater reliability where there was more than one respondent, future investigations should assess who and how to sample stakeholders. Additionally, the strategies can be interpreted differently by different stakeholders. For example “placing HCV medications on the formulary” was interpreted as a site-level strategy by some sites and not others, despite the fact that VA has a national formulary. While treatment starts were significantly and moderately correlated with strategies, other factors are likely involved in determining the number of treatment starts. These likely include patient factors and unmeasured site factors. It is also possible that sites employed additional implementation strategies that were not captured by the ERIC taxonomy or that they were not able to recall. Also, sites’ endorsements of strategies do not explain *how* the strategies were used or the extent of reach. While our methods did not allow us to understand the sequencing, intensity, or fidelity to each implementation strategy, the results demonstrate the feasibility of assessing a wide range of strategies nationally and the importance of strategy choice even in the context of a simple and highly evidence-based practice.

While this study is a first step towards understanding the role of strategy selection in clinical outcomes, there are several avenues for future research. Qualitative work could also help in determining the rationale for strategy choices and the perceptions of effectiveness. Future research should also assess the effectiveness of directing sites to specific strategies that have been thoughtfully selected based upon theory, evidence, and stakeholder input. In the past, implementation experts have advocated for systematic methods for selecting strategies based on evidence, theory, and stakeholder input [23]. Additionally an understanding of the cost effectiveness of specific strategies would certainly add to the field.

## Conclusions

This study is a first step towards better understanding how specific implementation strategies and clusters of strategies influence the uptake of a highly-evidence based, uniform innovation. The results demonstrate that the number of implementation strategies was associated with a meaningful clinical outcome. These results provide initial evidence for the clinical relevance of the ERIC strategies in a real-world setting on a large scale.

## Abbreviations

HCV: Hepatitis C virus
IQR: Interquartile range
VA: Veterans Affairs
